# Local Challenges and Successes Associated with Transitioning to Sustainable Food System Practices for a West Australian Context: Multi-Sector Stakeholder Perceptions

**DOI:** 10.3390/ijerph16112051

**Published:** 2019-06-10

**Authors:** Ros Sambell, Lesley Andrew, Stephanie Godrich, Justin Wolfgang, Dieter Vandenbroeck, Katie Stubley, Nick Rose, Lenore Newman, Pierre Horwitz, Amanda Devine

**Affiliations:** 1 School of Medical and Health Science, Edith Cowan University, Joondalup 6027, Australia; l.andrew@ecu.edu.au (L.A.); s.godrich@ecu.edu.au (S.G.); a.devine@ecu.edu.au (A.D.); 2 Perth Natural Resource Management, Perth 6104, Australia; justin.wolfgang@perthnrm.com; 3 Commonland, 103 Amsterdam, The Netherlands; dieter.vandenbroeck@commonland.com; 4 Centre for Social Impact, University of Western Australia, Crawley 6009, Australia; kathryn.dobb@uwa.edu.au; 5 Faculty of Higher Education, William Angliss Institute, Melbourne 3000, Australia; nick@sustainaustralia.org; 6 Sustain, The Australian Food Network, Melbourne 3000, Australia; 7 Geography and Environment, University of the Fraser Valley, Abbotsford, BC V2S 7M8, Canada; Lenore.Newman@ufv.ca; 8 Centre for Ecosystem Management, School of Science, Edith Cowan University, Joondalup 6027, Australia; p.horwitz@ecu.edu.au

**Keywords:** food system, food security, sustainable agriculture, regenerative agriculture, food system actors, challenges, successes, food supply

## Abstract

Large-scale food system practices have diminished soil and water quality and negatively impacted climate change. Yet, numerous opportunities exist to harness food system practices that will ensure better outcomes for human health and ecosystems. The objective of this study was to consider food Production, Processing, Access and Consumption domains, and for each determine the challenges and successes associated with progressing towards a sustainable food system. A workshop engaging 122 participants including producers, consultants, consumers, educators, funders, scientists, media, government and industry representatives, was conducted in Perth, Western Australia. A thematic analysis of statements (Successes (*n* = 170) or Challenges (*n* = 360)) captured, revealed issues of scale, knowledge and education, economics, consumerism, big food, environmental/sustainability, communication, policies and legislation, and technology and innovations. Policy recommendations included greater investment into research in sustainable agriculture (particularly the evidentiary basis for regenerative agriculture), land preservation, and supporting farmers to overcome high infrastructure costs and absorb labour costs. Policy, practice and research recommendations included focusing on an integrated food systems approach with multiple goals, food system actors working collaboratively to reduce challenges and undertaking more research to further the regenerative agriculture evidence.

## 1. Introduction

The food system encompasses the activities associated with producing, processing, distributing, purchasing and consuming food [1]. Food system drivers include agricultural productivity, resources such as land, water, labour, technology, as well as food consumption habits and food waste [1]. In turn, these food system activities and drivers influence food security, including availability, accessibility, affordability and desirability [2].

Food system drivers have resulted in practices that are negatively impacting climate change, land utilisation, water usage and pollution [3]. The resultant issues and challenges do not support a sustainable food system, specifically the soil integrity [4] necessary to ensure crop production meets the needs of the increasing global population [5]. Currently the agricultural sector has a strong dependence on fossil fuels, chemical fertilizers, pesticides and herbicides, which negatively impact waterways, soil and land, residues in food and ultimately human health [4]. Due to deforestation and poorly managed land use, there has been an exponential increase in soil erosion and concern in relation to feeding over 9.7 billion people by 2050 [6]. This will require food quantities produced over the last 500 years to be produced in the next 50 years [7], and is an impending food system challenge.

Internationally, the United Nations Development Programme’s Sustainable Development Goals (SDG) promote whole-of-society leadership, with “Goal 2” particularly focusing on sustainable food production and consumption, to increase the health of people and planet [8]. Governments facilitating sustainable agricultural provides the opportunity to foster SDG 2 for a food secure future.

Within the Australian context, food system challenges requiring urgent attention include the increasing foreign ownership of agricultural land, food distribution considerations, inadequate investment into Research and Development (R&D), and increasing debt and limited capital funds amongst producers [9]. There is also a dichotomy of inadequate food availability and quality for some of Australia’s population, yet excessive food waste amongst others. The lack of diversity between supermarket companies presents further distribution challenges for small-scale producers and retailers in the sector. These challenges, in addition to future issues such as unpredictable weather events, droughts and variability in temperatures, have further implications for food sustainability in Australia [9]. 

Western Australia (WA) is one of the seven jurisdictions within Australia, and geographically is isolated by vast distances from the greater population and food producing areas on the east coast of Australia. WA has a large agricultural region which has undergone massive land clearing and poor consecutive management which has adversely affected agricultural productivity of the land and now is recognized as one of 35 global biodiversity hotspots where ecosystems are at threat of extinction [10]. An increase in soil constraints and expenditure on farm inputs, and decreases in soil microbes, natural fertility, and farm gate and crop prices, has led to farmers scaling up operations, using innovation and new technologies to produce more food with fewer inputs, yet the challenges of climate change and diminishing natural resources limit capacity to produce enough food for Australia’s growing population [11]. Regeneration of soil, appropriate water catchment and management and maintenance of WA agricultural ecosystems are required to support and improve the WA agricultural industry. The WA economy is reliant on rural communities to produce adequate food for the population that is of sound nutritional quality to maintain health and food security. As a result this population is vulnerable due to their substantial reliance on interstate food freight [12], and in the event of environmental issues disrupting the food supply [13,14].

The Food Futures WA 2017 report, reinforced that Perth (WA’s capital city) lacked an economic development framework, which constrained use of available land for agriculture. This same report emphasized that intergenerational knowledge sharing was lacking and issues of scale were precluding further local food system progression [15]. Marslen [9] discusses how a reduction in soil and land degradation is fundamental to the sustainability of the current food system, and this requires amplified political commitment and leadership to reduce the impact of climate change on agriculture in Australia. These issues underscore the urgent need for transformative change to a more sustainable local food system. Proposed solutions to the aforementioned local food system challenges include increasing stakeholder engagement [16], aligning health systems, increasing economic resilience, increased support for R&D and regional agricultural investment [9]. Furthermore, The Food Futures 2017 report argued that consumer responsibility was required to ensure local food was more highly valued [15]. Consumer education on food choices and food waste were also considered important strategies to overcome a lack of consumer awareness [9]. Food system stakeholders in WA have also been urged to capitalize on technology, given its influence on consumer purchasing and this is seen as a strategy to increase connection between producers and consumers [15]. Expanding on the concept of connecting producers with consumers, it has been suggested to overcome issues of scale and for cooperative business models be developed, with producers collaborating to progress concepts such as ‘grower hubs’ that include production, processing and distribution of local food [15].

For the purpose of this research the term sustainable agriculture has been considered a collective for the practices used, albeit organic, biological, ecological or regenerative, that are being adopted by a movement of farmers in WA who identify as regenerative farmers. Regenerative agriculture includes increasing soil quality and greater carbon sequestering through no-tillage, use of cover crops, livestock and crop rotation [17,18]. By way of example, farm management techniques of a WA Wheatbelt farmer, who farms more than 13,000 ha of land, have produced competitive yields through sustainable farming processes which includes the “harnessing of the dynamic, natural relationships that exist between all the organisms in the ecosystem and the environment itself, particularly the soil” [19]. This farming method belongs to a breed of Australian regenerative farmers who, in Charles Massy’s terms, see their job as being “to get out of the way of Mother Nature” [20]. This sustainable, closed-loop method requires fewer farming inputs such as pesticides [21] and promotes collaboration across actors including scientists, farmers, consumers and decision-makers [22]. There is some evidence that adopting regenerative agricultural methods increases yield and productivity for specific crops [18]. However, WA research into regenerative farming practices is limited. There is much discussion about the urgency of the transition towards sustainable practices that preserve agricultural land, to support international agreements for reductions in greenhouse gas emission [23] and rising global temperature [24], yet there is an implementation lag. In principle regenerative farming methods are well supported by state government agencies [25], and can facilitate local solutions and foster land stewardship for future generations [26]. 

In order to progress the comprehensive food system change required to achieve local food security, a greater understanding of the challenges and successes associated with transitioning to sustainable food systems, in this context, is necessary [27]. Two underpinning concepts that support the framework for this research were used. The first is the food system expressed as Production, Processing and Consumption of food [27]. The second being the concept of food security whereby people have “physical and economic access to sufficient safe and nutritious food that meets their dietary needs and food preferences for an active and healthy life”, are underpinned by four dimensions: Access, availability, utilization and sustainability [28]. 

Therefore, the objective and novelty of this study were to explore the challenges and the successes associated with progressing towards a sustainable food system, in relation to food Production, Processing, Access and Consumption domains at a local level in WA. To the best of the authors knowledge this is the first WA study to incorporate a wide range of participant types to comment on the sustainability of the WA food system, therefore the emergence of these concepts has captured new information which can be shared and inform geographically relevant recommendations.

## 2. Materials and Methods

### 2.1. Design and Sample

This study used a qualitative methodology. The sample included 122 participants of a multi-sectoral workshop held in Perth, WA. Participants included media representatives, consultants, consumers, educators, funders, government representatives, industry, producers and scientists. Participants classified as media representatives promoted agriculture in the media; consultants included those participants self- or privately employed; consumers included those participants who possessed food knowledge; educators were associated with school or tertiary education, including academics; funders were classified as financial supporters of sustainable agriculture; government representatives worked in local, state or federal government; industry included stakeholders working in the agricultural sector but not for governments; producers were regenerative farmers, farm owners or food producers; and scientists/researchers were academic researchers. This diverse group of participants were invited to attend this workshop, given their role associated with food systems and given the importance of collectively harnessing expertise from a wide range of groups. A variety of recruitment methods were utilised. Workshop participants were identified through professional networks of the workshop organising team. This process utilised a non-probability method of recruitment recognised as Snowballing [29]. To overcome the potential of biased sampling and representation, purposive recruitment was also adopted to increase engagement with underrepresented sectors through an internet (Google) search. All participants were provided with the opportunity to opt out of the research.

### 2.2. Instrument and Data Collection

This qualitative study focused on open-ended responses placed on “post it” notes in response to two key questions. These questions related to key challenges and successes associated with progress towards a “local sustainable food system”, in relation to food Production, Processing, Access and Consumption domains. This framework allowed for the development of cross-cutting themes which is common to qualitative research that allows the analysis to respect the contribution of individual participants whilst capturing the complexity and nuances of their comments [30]. Quantifying the themes allowed the researchers to identify perceived successful practices reported by participants within the themes across the four domains and the challenges of sustainable farming across the same. Data were collected over the full-day workshop on 1 March 2018. 

### 2.3. Data Analysis

The research team included four mixed-methods researchers with experience in health, nutrition, education, food systems and food security, who collaborated with academics with expertise in agriculture, ecosystems and community development and engagement; industry experience in natural resource management and the built food environment; town planning, design and architecture; and international experience in regenerative farming. Thematic analysis was the chosen method for this research. This approach has been described as a “foundational method for qualitative analysis” and is well-regarded due to its flexibility [31]. The primary research team of four coders were each allocated one *a priori* domain to analyse: Food Production, Processing, Access or Consumption. As guided by the process outlined by Braun and Clarke [31], the coding process included six phases to create established, meaningful patterns. The phases included familiarization with data, generating initial codes, searching for themes among codes, reviewing themes, defining and naming themes, and producing the final report. The first step, familiarisation with data, included all coders reading raw statements within their allocated domain, noting possible patterns within data. Data were then transcribed verbatim into Microsoft Excel and subsequently imported into QSR NVivo version 12 (QSR International, Melbourne, Australia) [32]. Once coders had familiarised themselves with the content of their respective domains, they began generating initial codes. 

The second step, generating initial codes, involved coders generating collectively the overarching “parent codes” within NVivo, including the food system domain names (i.e., food production), with the “child codes” being data driven. The inductive codes were named according to the content contained within them. Coders generated notes to document key reflections, queries that required team discussion and consensus, and a description of how data were interpreted. Each coder devised a table, which included the code name, a description of the code’s key concepts and an exemplar quote. Parallel coding (coding data into as many codes as relevant) was conducted where appropriate [33]. Code descriptions were re-read and cross-checked with individual coded statements. Code descriptions within NVivo were updated where relevant, to ensure the breadth of topics within each code was captured. 

The third step included searching for themes, through the process of cross-checking all coding within the child codes, combining or separating codes where required and discussing the reorganisation of codes into broader themes. The subsequent “reviewing themes” phase [31] involved further refining and combining themes. The coding team reviewed the statements within each theme to ensure data contained within formed a defined pattern. In addition the review process allowed the narrative of the dataset as a whole to be established [31]. 

The penultimate step included defining and naming themes (individually and the dataset as a whole) and compiling a thematic description for each theme. Relevant sub-themes were also identified where appropriate and final theme names were decided. The last data analysis step, producing the final report, included the preparation of each theme’s description with exemplar quote(s) to illustrate the concepts within each theme [31].

### 2.4. Ethical Approvals

Ethical approval was provided by the Edith Cowan University Human Research Ethics Committee (project number 19953).

## 3. Results

Table 1 outlines participant demographics. The most common participant type, represented by 27% of attendees, was a food producer, followed by an educator (15%). The least common participant type in attendance was a funder, representing only 2% of workshop attendees.

Participant statements were coded and clustered by theme and intent (Successes (*n* = 170) or Challenges (*n* = 360)), with the number of references allocated to Production, Processing, Access or Consumption domains. As demonstrated by Table 2, food system actors most frequently discussed challenges associated with themes such as Issues of scale (*n* = 89 coded statements), Knowledge and education (*n* = 66), and Economics (*n* = 63). Across all themes, the Production domain presented the most challenges (*n* = 110), particularly with respect to Issues of scale (*n* = 33), Economics (*n* = 33) and Knowledge and education (*n* = 19) themes. Consumption was the second most prevalent domain where the Consumerism theme (*n* = 44) was seen as a challenge, followed by the Knowledge and education theme (*n* = 32). Access was the third highest ranked domain based on the number of coded statements (*n* = 78), with Issues of scale and Knowledge and education the top two most coded themes with respect to challenges. The Processing domain had the least coded statements (*n* = 74), with Issues of scale (*n* = 34) and Economics (*n* = 20) as the greatest challenges. Only one participant considered Technology a challenge, citing an unreliable internet as a challenge to communication processes within food production. In contrast, however, the Technology domain was most strongly associated with success, most notably in production (Table 3).

Participants also outlined successes associated with transitioning to a sustainable food system (Table 3). According to respondents, the greatest area of success was in Technology and innovation (*n* = 61 coded statements), which was most prevalent in Production (*n* = 40) and Processing (*n* = 18) domains. Consumerism, Issues of scale and Knowledge and education were three similarly represented themes for successes. However, Consumerism was more strongly represented in Consumption and Processing domains. Issues of scale references were more common in Processing and Access domains. Knowledge and education successes were coded in Consumption (*n* = 18), Access (*n* = 8) and Production (*n* = 5) domains. Environmental/sustainability, Communication, Economics, Policies and legislation had few coded statements with respect to successes. Further to this, successes associated with “Big food” were not reported in any of the domains by participants.

In order to elicit the key issues, from participants responses, data were further interrogated, resulting in the identification of well-established cross-cutting themes and narrative of challenges and successes in this current state of play. The themes were applicable across one or more Production, Processing, Access and Consumption domains but may not have featured as both a challenge and a success. Table 4 outlines the overarching themes and more detail of embedded sub-themes, and provides exemplar coded statements.

### 3.1. Cross-Cutting Themes and Sub-Themes

#### 3.1.1. Big Food

The term “big food” is an umbrella term to denote the leading powers in the current food system, in particular large agribusinesses and suppliers. Participants exclusively regarded the theme of big food as a challenge to transitioning towards a sustainable food system. Big food was perceived to have appreciable power over food availability, with significant marketing and advertising power playing an influential role in consumer food choices. Similarly, large-scale agribusiness was believed to have market control, which led to a perceived corporatisation of farming.

#### 3.1.2. Communication

Participants’ perception of communication focused on technology and miscommunication. Communication was almost entirely linked to the domain of Access. Within the sub-theme of technology, poor internet availability and reliability were a commonly highlighted as factors limiting communication between consumers, producers and processers. Few participants reported that improvements in this area had supported sustainable food system success. Poor or miscommunication between various stakeholders, in particular producers and researchers, was seen as a further challenge. Issues of mistrust and a lack of shared language were also identified. Reflecting the communication technology findings, a few participants identified improvements in this area as a success, most notably between producers and consumers.

#### 3.1.3. Consumerism

Considered a challenge across all four domains, the theme of Consumerism was divided into the sub-themes of consumer expectations and consumer practice. Consumer expectations were seen to be at odds with sustainable food systems and included the demand for ready-made meals and cheap and out-of-season produce. Unhealthy consumer practices, such as low fruit and vegetable intake, and high consumption of processed foods were considered the norm, and were identified as further challenges. A positive shift in consumer expectation was noted by other participants and included increasing demand for healthy, local and sustainable food options, which were noted as successes. Other successful changes in consumer practices included dietary diversification, greater use of vegetables such as “ugly veg”, and shopping for local produce. 

#### 3.1.4. Economics

Participants almost entirely perceived economic issues as sustainable farming challenges. The rural setting was associated with a range of financial challenges, including relatively high cost of fresh produce and lack of rural distribution centres. Further, food processing and availability in rural areas were influenced by high transport and production costs. High labour costs, and expectations of local workers, was seen as additional economic burdens to local processing and production. Capital costs, such as the cost of equipment needed for producing, processing and access (transporting goods), were seen as further limiting factors. This was viewed as potentially prohibitive for smaller-scale businesses. Successes in this area were uncommon, and were restricted to the Access domain and related to the subject of investment in regenerative agriculture.

#### 3.1.5. Environmental Sustainability

Sub-themes regarded as challenges were natural resources and weather/climate change. Soil degradation and poor soil quality were seen as issues affecting production, as was an unreliable supply of water in terms of quality and quantity. The weather sub-theme revealed drought, flood and bush fire challenges. The impact of climate change on weather events and natural resources was a concern reported by two participants.

#### 3.1.6. Knowledge and Education 

The sub-theme, “gaps in available evidence” to support sustainable farming, was seen as a challenge. “Gaps in knowledge” among consumers was a further sub-theme. Poor health literacy and “ecological literacy” were identified as challenges. In particular, insufficient understanding of food preparation and production, seasonality of food, nutrition content, and food origins were noted. “Gaps in accessibility of knowledge” was a further important challenge. A common issue was the inadequacy of the Australian food labelling system to inform the public of fresh food provenance and nutritional value.

Successes in this theme were evident across the domains of Access, Production and Consumption. A number of practices were described that supported knowledge sharing, such as community workshops and kitchens. Better links between the growers’ market, producers and processors of food were also seen as a step towards more successful knowledge sharing. “Emerging evidence” to support sustainable agriculture included the importance of microbes (gut health) and the potential harm associated with heavy metals in produce.

#### 3.1.7. Issues of Scale

The small-scale nature of sustainable farming, in terms of production, processing and accessibility of produce was a strong theme that posed a number of different challenges. A lack of local infrastructure with regards to equipment, local labour and storage were seen as problematic, as was the lack of guaranteed availability of a product to meet market demand and paying ongoing costs. Economies of scale were also viewed as important; that is, the difficulty in justifying the cost associated with buying equipment necessary in small-scale operations was raised. Successful practices to overcome or alleviate these issues of scale involved collaboration, such as small-scale home businesses (cottage industry alliances) and shared commercial kitchens for value-added production.

#### 3.1.8. Policy and Legislation

Existing policies and legislation were seen as a challenge to sustainable agriculture. There was a perception among many participants that inadequate government investment and support was provided for “regenerative farming”. The term “red tape” was commonly used across this theme. Successes were limited to Processing; one example provided was local abattoir practice. Land use laws were seen as both a challenge (poor access to land) and a success (cheap land in WA). 

#### 3.1.9. Technology and Innovation

Although unreliable at times, especially in regional and remote WA, the internet was almost overwhelmingly viewed an important mechanism to information and practice sharing between stakeholders. The emerging agriculture-based robotic technology was also seen as a potential area for success. No discussion was made of other potentially supportive technology, such as farm software solutions or artificial intelligence.

Whilst the potential risks associated with investment were seen as a challenge to future innovation, many examples of innovative practices were identified. These included changes to packaging, re-use and reduction of waste, urban and school gardens and the emerging bush-tucker market.

## 4. Discussion

The study found that the most frequent food system challenges were associated with Production, followed by Consumption, Access and Processing domains. The highest frequency of successes was equally reported within the Production and Processing domains, followed by the Consumption and Access food system areas. The high representation of challenges in the Production domain may relate to the slight overrepresentation of producers in the workshop. However, all participants had a vested interest in the food system, also as consumers, and had a voice that was captured. Throughout the workshop there was much discussion between participants, producers and consumers about the “how” of transformation and a strong narrative emerged about addressing the urgency and implementation lag, and this can be seen in the successes that emerged in the domains of Production and Processing especially. 

Cross-cutting themes identified included: Issues of scale; Knowledge and education; Economics; Consumerism; Big food; Environmental/sustainability; Communication; Policies and legislation; and Technology and innovations. The perception of inadequate government investment and support of regenerative farming was a challenge reported by participants, which was compounded by gaps in available evidence to support regenerative agricultural practices. Within WA there has been demonstrated evidence from governmental bodies of support for sustainable agricultural practices. However, these findings suggests the method of communication between supporting government bodies and sustainable agriculturalists needs addressing [34]. Participants in this study focused on inadequate government support for sustainable farming and associated “red tape” that increased implementation lag. Food system actors that were interviewed to shape the Foodprint Melbourne report highlighted policy issues such as the siloed, piecemeal approach to food systems [35] that similarly slowed action. Furthermore, inadequate local infrastructure, such as equipment, local labour and storage, further precluded progress towards sustainable food system practices, which is consistent with other studies [36]. 

Economic challenges included the high cost of transporting and accessing fresh produce which was heightened by a lack of rural distribution centres. The power exerted by large multinational businesses over food availability and advertising was highlighted, as was a lack of consumer knowledge in areas of Production, Access and Consumption, which potentially led to consumer expectations being “at odds” with sustainable food systems. Lusk and McCluskey [37] suggest that agricultural economists can play a significant role in addressing complex issues relating to food demand, as they can provide tools and insights to address the required changes in food consumption patterns which could see the proliferation of consumer demand increasing the demand for food from sustainable agriculture practices.

An additional challenge reported was environmental factors, and included soil degradation and an unreliable water supply which confounded poor soil quality that was evident in current farming practices, potentially affecting the ease and cost of transition to sustainable agriculture. Grover and Gruver [36] conducted place-based research in the United States, to explore the slow adaptive process of sustainable farming and found poor soils contributed to a slower uptake of these practices. There is also evidence of studies in Africa that discuss more sustainable water management practices and recommends that governing institutions provide more guidance and work with producers to ensure sustainable practices are embedded and maintained to foster local water security [38]. It is well recognised that in the Australian context, water and soil security should be prioritised and that Australia is ill prepared to manage the effects of climate change [39]. A report completed by Lockie [39] suggests that climate mitigation policies would positively impact Australian agriculture and are in urgent need of consideration and implementation.

This research was also able to identify perceived successes from participants, and these were associated with utilisation of improved technology, which enhanced stakeholder communication, investment in regenerative agriculture and the low land costs in some WA areas. Knowledge-sharing and collaboration between food system actors were additional assets discussed to extend reach and action. Technology utilisation in the agriculture sector is widespread from, but not limited to, soil sampling kits, weather predictions, driverless equipment and web-based communication and technology. This could be seen as “par for the course” as part of industrialization. However, it can hide social implications for rural communities including a trend to reduced employment [39]. The increasing median age of current farmers is coupled with reduced inter-generational skill transition and the inability of farms to support more than one family, escalating rural depopulation [39]. Solutions suggested by this same author include the protection of small land holdings external to more populated rural areas, as small land holdings have less entry barriers and contribute to local food security. In addition, research conducted by Salim, et al. [40] suggests that communication technologies positively affect agricultural output in the long run, and government policies should facilitate investment in telecommunication facilities to support agriculture. Improved technology has been cited as a success in previous research, such as improved irrigation technology that increases yields and reduces water requirements [41]. The narrative acknowledged the emerging and positive role of agricultural technologies in future food production. Similarly, previous research has suggested that information technology holds the potential to support sustainable agriculture through tailoring required inputs [42]. In addition, these technologies also hold potential for post-harvest processing, access and consumption stages, thus increasing the sustainability of the food system [42]. Gregor [43] found that, in general, Australian farmers were positive about computers and the internet and they considered technology to help them in their role.

The positive shift in consumer expectations was seen as a success, exemplified by the increasing demand for healthy, local and sustainable food options among some population segments. There is evidence that consumers are demonstrating a preference for quality differentiated products, albeit organic, locally grown, sustainably grown and free from genetic modification, and there is increasing evidence that consumers value these product claims [44]. Palma, Collart and Chammoun [44] emphasise that information communicated to consumers has to be succinct and truthful to avoid confusion, which supports the participants views across the domains regarding the lack of a shared language for better communication between all actors. Knowledge and education were cited as a challenge and success by our participants, and increased connection between producers and consumers was highlighted as a successful strategy to progress a sustainable food system agenda. This is supported by existing evidence [35] and highlighted opportunities to connect producers with consumers, including direct sales, conduct agri-tourism activities and creation of educational consumer experiences. 

Furthermore, innovation across many parts of the food system were apparent, with re-use and reduction of waste and the emerging bush-tucker market cited as successes. This is also supported by current international research [44], where consumers are demonstrating a readiness to accept “native” products especially in light of improving food security through indigenous food variety and biodiversity [39].

These workshop findings add substantial value to the sparse evidence base in WA on this topic. The Food Futures WA 2017 report reinforced that Perth lacked an economic development framework, constraining use of available land for agriculture [15]. The Policy and legislation theme emphasised challenges associated with land use laws, while the Economics theme included discussion of challenges associated with rural access to local food. The Food Futures 2017 report argued that consumer responsibility was required to ensure local food was highly valued [15]. The workshop discussions reflected this finding, as there were reported perceptions of a shift in thinking among other population segments, representing an opportunity that could be harnessed. This reinforces the importance of consumer education and broader knowledge translation from food system actors through strategies such as improved food labelling, concise messaging and a common language. Food system stakeholders in WA have been urged to capitalise on technology, given its influence on consumer purchasing [15]. These findings support this, with workshop participants outlining how technology enhanced such relationships, particularly robotic technology seen as a success, but there was no discussion of other potentially supportive technologies, such as farm software solutions. Issues of scale were raised, with cost constraints experienced by small-scale producers suggested to be overcome by collaboration between producers. Previous WA work has similarly recommended cooperative business models as a strategy to overcome pricing constraints [15].

These findings are also relevant in a national and global context. They both compared and contrasted with the existing evidence base, albeit most other studies have used different methods to that presented here and some were in other countries so lacking the local context. Previous evidence highlighted high food production costs and price volatility as key threats to sustainable agriculture. Strategies to overcome these challenges included increased use of information and communication technology, government policies supporting trade, storage and price stabilisation [41]. These results relating to economics did not focus on market price volatility, but highlighted the need for greater capital funds required by farmers to produce, process and transport food. Other Australian research suggested the high costs borne by farmers to produce and transport food was a key challenge to farm viability [35] and in part related to the high cost of labour being a key food production challenge [35]. The current dysfunctional food system has been discussed by others in similar ways to our study participants, with discourse suggesting a rapid increase in health issues whilst negatively impacting the environment [45].

Unsustainable food production and consumption patterns have been labelled “among the most important drivers or environmental pressures” [45]. Previous research has well and truly established the links between unsustainable agricultural practices and soil erosion, degradation and waterlogging, exacerbated by unpredictable weather patterns [41]. Successes reported in our study that supported the existing evidence base included increased soil fertility through reduced reliance on fertilisers and incorporation of nitrogen-fixing crops [41]. Interestingly, aspects of practices with an increased emphasis in mainstream agriculture, which are embraced by (and probably originate from) the sustainable agriculture discourse, and concomitantly included by proponents of regenerative agriculture, were missing from the dialogue we recorded. These included minimum tillage and steps taken towards soil conservation. While workshop discussants recognised an increasing interest in organic carbon and soil health, it could not be developed as a theme. These aspects might be best explained by a low proportional representation of (environmental and government) scientists in our stakeholder groups. Similarly, the social movement of Landcare and other “organic” enablers (from biodynamics to consumer advocacy and farmer’s markets) were not explicitly captured as successes.

Numerous factors were highlighted by this and previous research in relation to achieving sustainable food system practices. A systems-based approach is required that considers the complexity of the issue [45]. Consequently, recommendations from this study have implications for policy, practice and research and were determined by this study’s results and supported by existing evidence. 

Extrapolated from this study, future policies for sustainable food systems would be integrated to consider multiple cross-sectorial goals, radicalizing efforts in land preservation, encouraging greater investment into regenerative agriculture research, supporting producers to overcome high infrastructure costs and absorb labour costs, strong government risk mitigation policies and facilitating inclusion of multi-sector stakeholders in the food system dialogue, which includes a focus on government agencies improving their communication pathway and subsequent support with these stakeholders [45,46]. These recommendations would support ecosystem approaches to agricultural practices that prioritise soil, water and land protection, reduce economic challenges faced by producers, consider multiple perspectives in decision making and would demonstrate strong leadership. In the process an evidence base would be built. None of these things will be easy or possible unless the influence of the neoliberal socio-political driver is concomitantly addressed.

Practice-based implications include food systems actors supporting sustainable food production, such as reinforcing local food production and processing, reducing food waste [45], strengthening long-term partnerships and collaboration to enhance knowledge-sharing and reduce infrastructure costs, harnessing information technology, and focusing on increasing consumer demand for healthy, local, seasonal food [46]. This could be facilitated by producer–consumer educational experiences, delivered through community and school settings. Research recommendations include exploration of interactions within food system levels *and* between the food system and other systems (such as technological development) [46].

This study’s strengths include the inclusion of multi-sector stakeholders as participants, increasing the diversity in responses received across food system domains. Multi-sector stakeholder contribution and knowledge sharing has been cited as vital to understand food system processes [46]. Further, the workshop fostered collaboration and enabled cross-sectoral dialogue, often perceived as approaches to wicked problems of this sort. Data collection over a whole-day workshop, as opposed to data collected via other methods, enabled ideas to be explained and interpreted collectively, with participant checking taking place. In addition, the presentation of cross-cutting themes in this study exemplifies the multiple intervention points available to promote sustainable change. This study is not without limitations. Data were collected during a one-day workshop, with anonymous responses which did not enable researchers to cluster comments by participant type to provide a more in-depth interpretation. Not only did this data collection approach limit the quantity and depth of available statements from which to draw on, it precluded participant follow-up to clarify any statements. The Processing and Access domains had a lower frequency of coded statements, which could reflect the participant types that attended the workshop. The workshop attendees’ sectors were unequally represented, for example, the manufacturing or processing sector was not well represented, nor was government and scientist as mentioned above. Finally, the research team’s interpretation of the intent behind these brief statements and subsequent coding based on this, are further potential limitations. 

## 5. Conclusions

This study highlighted various food system actor perceptions of the challenges and successes associated with transitioning towards a sustainable food system, across Production, Processing, Access and Consumption domains. The research highlighted that whole-of-system leadership is required to ensure the ecosystem functions, processes and services upon which the health of the planet and its people are based. Policy, practice and research recommendations included focusing on an integrated food systems approach with multiple goals, food system actors working collaboratively to reduce challenges and undertaking more research to further the regenerative agriculture evidence base. In order to achieve the United Nation’s Sustainable Development Goals, all food system actors must work collectively to transition towards sustainable food production practices.

## Figures and Tables

**Table 1:** Participant demographics.

Participant Type	Number of Participants (%)
Media	6 (5%)
Consultant	11 (9%)
Consumer	11 (9%)
Educator	18 (15%)
Facilitator	10 (8%)
Funder	2 (2%)
Government	12 (10%)
Industry	15 (12%)
Producer	33 (27%)
Scientist	4 (3%)
TOTAL	122 (100%) *

* A number of participants were categorised into more than one participant type.

**Table 2:** Challenges associated with transitioning towards a sustainable food system, across domains and themes determined by participants.

Theme	Challenge Total	Domain			
		Production	Processing	Access	Consumption
Issues of scale	89	33	34	22	0
Knowledge and education	66	19	0	15	32
Economics	63	33	20	10	0
Consumerism	57	3	4	6	44
Big food	49	11	11	6	21
Environmental/sustainability	12	9	0	3	0
Communication	10	1	0	8	1
Policies and legislation	13	0	5	8	0
Technology and innovations	1	0	0	0	0
**TOTAL**	**360**	**110**	**74**	**78**	**98**

**Table 3:** Successes associated with transitioning towards a sustainable food system, across domains and themes determined by participants.

Theme	Success Total	Domain			
		Production	Processing	Access	Consumption
Technology and innovations	61	40	18	3	0
Consumerism	33	0	15	0	18
Issues of scale	32	9	13	10	0
Knowledge and education	31	5	0	8	18
Environmental/sustainability	6	0	6	0	0
Communication	3	0	0	3	0
Economics	2	0	0	2	0
Policies and legislation	2	0	2	0	0
Big food	0	0	0	0	0
TOTAL	170	54	54	26	36

**Table 4:** Cross-cutting themes, embedded sub-themes and exemplar statements.

Theme	Subtheme	Challenge or Success (C or S)	Examples of Direct Quotes
**Big food**	**The power of supermarket chains for economic gain**	**C**	These organisations drive the market/control availability of foods Monopolisation of Coles, Woolworths—lots of control
	**Impact of marketing and advertising by big organisations**	**C**	People attracted by brands not quality/nutritional value Misinformation due to marketing campaigns
	**Big agribusiness control over food markets**	**C**	Corporatisation of farming Industrial agriculture—privilege, big business/farms
**Communication**	**Communication technology**	**C**	Poor internet Better internet needed
		**S**	Internet opportunities to improving communications
	**Communication and miscommunication**	**C**	Need shared language for better communication between farmers and researchers Gap between farm and research institute (lack of trust in farmers, different languages)
		**S**	Better communication between local farmers and local community (connection of farmer with consumer)
**Consumerism**	**Consumer expectations**	**C**	Consumers want convenience/ [they are] time poor Consumer demand for unseasonal produce
		**S**	Slow food movement and demand for sustainable practice in society People are demanding better food
	**Consumer practices**	**C**	Food as reward rather than health resource General public not consuming enough fruit and vegetables
		*S*	Cultural diversity/diverse diets Using vegetable differently (cauliflower rice)
**Economics**	**Rural location**	**C**	High cost of healthy foods in remote locations Centralised distribution centres—reduces rural access to local food Food miles
	**Labour**	**C**	Labour costly High wage expectations
	**Capital costs**	**C**	Capital for new farmers to grow
		**S**	Impact (sic: of) investment in regenerative farming/agriculture Capital—new farmers, small scale farmers to grow and value add Government ease of access to support (grants)
**Environmental sustainability**	**Weather**	**C**	Environmental instability (fire, storms etc.) Climate change
	**Natural resources**	**C**	Drought/water access Soil quality
	**Waste reduction**	**S**	Waste recycling/repurposing (e.g., coffee grounds) Biodegradable packaging
**Knowledge and education**	**Evidence gaps**	**C**	Research and development Lack of research on regenerative farming
	**Knowledge gaps**		Poor food literacy—where and how food is produced Strategic thinking and oversight about how to enable alternative processing
	**Accessibility of knowledge**	**C**	[Inadequate] Food labelling system Knowledge [of] products for producers—hard to access/find relevant info
		**S**	Workshops–produce development (Example-Serpentine Jarrahdale Food Alliance) Gaining linkages to those with market expertise (link producers to market experts)
	**Emerging evidence**	**S**	Microbes awareness Chemical/heavy metal residues detected in produce achievable
**Issues of scale**	**Infrastructure**	**C**	Critical mass for infrastructure and equipment Logistics of selling, storage, distribution and marketing
	**Collaborative practice**	**C**	Sustainability of supply [from small-scale producers]
		**S**	Cottage industries collaborating Shared commercial kitchens (e.g., Far Harvest Perth)
**Policies and legislations**	**Legislative environment**	**C**	Lack of government direction Red tape
		**S**	Farm abattoir- change in legislation Government policy
	**Land use policy**	**C**	Access to productive land
		**S**	Cheap land in Western Australia
**Technology and innovation**	**Technology**	**C**	Issues with internet access
		**S**	Innovation through robotics Technology—communicating new information
	**Innovative practice**	**C**	Risk of changing practice (money, time, health) sits with farmer
		**S**	Ugly food, cardboard tomato boxes, local abattoirs, urban beekeeping, urban farming urban rooftop gardens, school gardens
